# An alternative to force

**DOI:** 10.7554/eLife.08659

**Published:** 2015-06-05

**Authors:** Amanda Patel, Sophie Demolombe, Eric Honoré

**Affiliations:** Institut de Pharmacologie Moléculaire et Cellulaire, Valbonne, France; LabEx ICST, Valbonne, France; UMR 7275 CNRS, Valbonne, France; Université de Nice Sophia Antipolis, Valbonne, Francehonore@ipmc.cnrs.fr

**Keywords:** ion channel, agonist, mechanotransduction, red blood cells, physiology, cell volume regulation, mouse

## Abstract

Researchers have discovered a synthetic small molecule that activates a mechanosensitive ion channel involved in a blood disorder.

**Related research articles** Syeda R, Xu J, Dubin AE, Coste B, Mathur J, Huynh T, Matzen J, Lao J, Tully DC, Engels IH, Petrassi HM, Schumacher AM, Montal M, Bandell M, Patapoutian A. 2015. Chemical activation of the mechanotransduction channel Piezo1. *eLife* **4**:e07369. doi: 10.7554/eLife.07369 Cahalan SM, Lukacs V, Ranade SS, Chien S, Bandell M, Patapoutian A. 2015. Piezo1 links mechanical forces to red blood cell volume. *eLife* **4**:e07370. doi: 10.7554/eLife.07370

**Image** Yoda1 causes red blood cells to shrink (top), but not when the gene for the Piezo1 ion channel is knocked out (bottom)

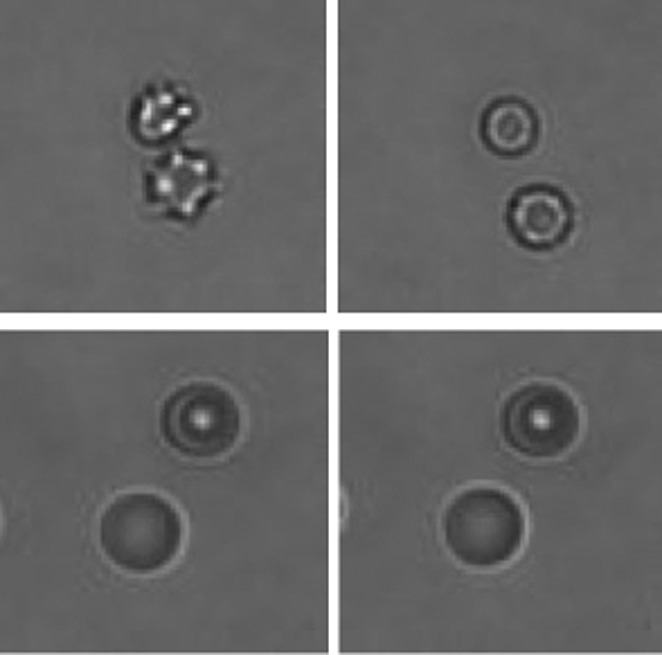

Ion channels are transmembrane proteins that allow ions to move in or out of cells, and they are vital to a range of biological processes. They can be opened and closed in a number of ways: for example, some are opened by voltage, while others respond to the binding of ligands. Piezo1 and Piezo2 are mechanosensitive ion channels: in other words, they open in response to mechanical stimulation, such as stretching or shear stress (Coste et al., 2010, 2012).

Mutations in the gene *Piezo1* have been linked to a blood disease called xerocytosis that leads to hemolytic anemia (Albuisson et al., 2013; Bae et al., 2013; Coste et al., 2013; Zarychanski et al., 2013). It is known that these mutations reduce the ability of the Piezo1 ion channel to close, and this leads to red blood cells shrinking as a result of dehydration. However, the details of this process are not fully understood. Now, in a pair of papers in *eLife*, Ardem Patapoutian, Michael Bandell and colleagues at the Scripps Research Institute, the Genomics Institute of the Novartis Research Foundation and the University of California San Diego show that the Piezo1 ion channel can be opened in the absence of mechanical stimulation by a synthetic small molecule called Yoda1. This, in turn, results in dehydration via the secondary activation of a different ion channel.

Piezo1 is expressed in the endothelium of developing blood vessels, making them sensitive to shear stress: in particular, if the flow of blood through a blood vessel increases, the Piezo1 ion channel opens and calcium ions move into the cells of the endothelium (Li et al., 2014; Ranade et al., 2014a). Similar ion channels are found in invertebrates, which suggests that this mechanism for the transduction of mechanical forces into biological responses is conserved (Coste et al., 2010). Piezo2 is expressed in the sensory nervous system and has a central role in the sense of touch (Maksimovic et al., 2014; Ranade et al., 2014b; Woo et al., 2014).

The first of the Piezo1 papers—which includes Ruhma Syeda as first author—reports the findings of a screen of over three million synthetic small molecules that searched for molecules that could activate the Piezo ion channels (Syeda et al., 2015). This screen identified a molecule that could activate Piezo1 (but not Piezo2). Yoda1 contains two chlorines and a thioether group, which are both essential for its activity. At micromolar concentrations, Yoda1 made Piezo1 much more sensitive to mechanical stimulation, and also slowed down the inactivation of the ion channel.

Yoda1 was also able to open Piezo1 ion channels that had been inserted into an artificial membrane, without the presence of other proteins or the application of any mechanical stimulation. Further experiments revealed that Yoda1 mainly acts to stabilize the ion channel in its open state. These findings suggest that Yoda1 may act directly on Piezo1 and/or on the membrane, although it seems unlikely that it acts on the membrane because Yoda1 does not activate Piezo2. This finding is important because it suggests that an equivalent molecule might exist in nature and could possibly open Piezo1 in the absence of mechanical stimulation.

The second paper—which includes Stuart Cahalan and Viktor Lukacs as joint first authors—took advantage of the properties of Yoda1 to explore why activation of the Piezo1 ion channel causes red blood cells to shrink (Cahalan et al., 2015). They started by demonstrating that Piezo1 is expressed on both peripheral mature red blood cells and on pro-red blood cells developing in bone marrow in mice. Deletion of the *Piezo1* gene in the hematopoietic system was used to decipher the functional role of the Piezo1 ion channel in red blood cells.

In brief, they found that the opening of Piezo1 by Yoda1 causes red blood cells to shrink via the secondary activation of another ion channel, the KCa3.1 Gardos channel, by calcium ions that enter the cell through the open Piezo1 ion channel. This leads to potassium ions moving out of the cell (via the Gardos channel) and a consequent loss of water (Figure 1). On the other hand, red blood cells without Piezo1 ion channels were overhydrated: they also had an increased osmotic fragility, were enlarged in size, and tended to be retained in the spleen. These findings suggest that Piezo1 is important for the integrity and recirculation of red blood cells.

**Figure 1.**
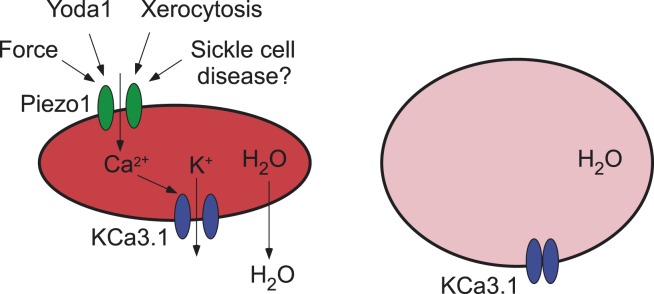
Ion channels and red blood cells. (**Left**) Opening the Piezo1 ion channel (green) by mechanical stress or by the synthetic small molecule Yoda1 promotes an influx of calcium ions that stimulates the opening of the KCa3.1 Gardos channel (blue). The resulting efflux of potassium ions through the KCa3.1 Gardos channel leads to a loss of water from the red blood cell, which causes it to shrink. Mutations in the *Piezo1* gene reduce the ability of the Piezo1 ion channel to close, which causes red blood cells to shrink in the disease xerocytosis. Excessive opening of the Piezo1 ion channel might also be involved in sickle cell disease. (**Right**) When the gene for Piezo1 is deleted, there is no influx of calcium ions, so the KCa3.1 Gardos channel remains closed and the cell becomes overhydrated. Red blood cells that lack the KCa3.1 Gardos channel also become overhydrated (not shown; Grgic et al., 2009).

The work of Patapoutian, Bandell and co-workers is important because it demonstrates how mechanical forces could lead to the shrinkage of red blood cells. But why would red blood cells shrink in response to force? One possibility is that it might improve their ability to pass through small capillaries and/or it might allow hemoglobin to be concentrated within red blood cells, thus promoting the release of oxygen.

Previous work has suggested that the opening of stretch-activated ion channels might contribute to the sickle cell disease, which is caused by red blood cells with abnormal sickle-like shapes accumulating in capillaries (Ma et al., 2012). It is tempting at this stage to propose that the opening of Piezo1 ion channels might contribute to the altered ionic homeostasis that is seen in red blood cells in sickle cell disease. It should be possible to test this idea by studying mice in which the *Piezo1* gene has been conditionally deleted and in which the hemoglobin carries the sickle cell mutations. If this hypothesis is indeed verified, it might be possible to use molecules that can inhibit Piezo1 ion channels to treat a disease that affects millions of people, mostly in sub-Saharan Africa.
